# Stem Cells in the Treatment of Neuropathic Pain: Research Progress of Mechanism

**DOI:** 10.1155/2020/8861251

**Published:** 2020-12-28

**Authors:** Meichen Liu, Kai Li, Yunyun Wang, Guoqing Zhao, Jinlan Jiang

**Affiliations:** ^1^Department of Anesthesiology, China-Japan Union Hospital of Jilin University, Changchun, China; ^2^Department of Scientific Research Center, China-Japan Union Hospital of Jilin University, Changchun, China

## Abstract

Neuropathic pain (NP) is pain caused by somatosensory nervous system injury or disease. Its prominent symptoms are spontaneous pain, hyperalgesia, and allodynia, and the sense of pain is extremely strong. Owing to the complex mechanism, conventional painkillers lack effectiveness. Recently, research on the treatment of NP by stem cells is increasing and promising results have been achieved in preclinical research. In this review, we briefly introduce the neuropathic pain, the current treatment strategy, and the development of stem cell therapy, and we collected the experimental and clinical trial articles of many kinds of stem cells in the treatment of neuropathic pain from the past ten years. We analyzed and summarized the general efficacy and mechanism of stem cells in the treatment of neuropathic pain. We found that the multiple-mechanism approach was different from the single mechanism of routine clinical drugs; stem cells play a role in peripheral mechanism, central mechanism, and disinhibition of spinal cord level that lead to neuropathic pain, so they are more effective in analgesia and treatment of neuropathic pain.

## 1. Introduction

Pain is the body's response to external injury or internal disease. Normal pain is essential to an individual's risk perception and hazard avoidance [1]. Chronic pain is defined as pain that persists or recurs for more than 3 months [2]. Its prevalence rate is about 11 to 19% of the adult population [3–5]. Neuropathic pain (NP) is pain caused by injury or disease of the somatosensory nervous system [6, 7], which accounts for 20 to 25% of patients with chronic pain; its prevalence rate in the general population may be as high as 7 to 8% [8]. Despite a high prevalence of NP, there is a lack of effective treatment for NP in modern medicine. As a novel treatment, stem cell therapy has achieved remarkable results in the preclinical study of NP.

## 2. Classification, Clinical Manifestations, and Diagnosis of Neuropathic Pain

Neuropathic pain is defined as pain caused by damage or disease of the somatosensory nervous system [9, 10]. This kind of pain is usually observed in the innervated area of the body with a damaged nervous system structure (projection pain) [2]. In 2019, the International Association for the Study of Pain (IASP) made a detailed classification of NP, dividing it into chronic peripheral neuropathic pain and chronic central neuropathic pain [11]. Chronic peripheral neuropathic pain is caused by pathological changes or diseases of the peripheral somatosensory nervous system, which mainly includes trigeminal neuralgia, chronic neuropathic pain after peripheral nerve injury, painful polyneuropathy, postherpetic neuralgia, and other specified and unspecified chronic peripheral neuropathic pain, while chronic central neuropathic pain is caused by central somatosensory nervous system damage or diseases, including chronic central neuropathic pain associated with spinal cord injury, chronic central neuropathic pain associated with brain injury, chronic central poststroke pain, chronic central neuropathic pain caused by multiple sclerosis, and other specified and unspecified chronic central neuropathic pain.

Unlike nociceptive pain, NP is typically characterized by positive (enhanced somatosensory function) and negative (loss of somatosensory function) sensory symptoms and signs, including burning pain, evoked pain, and abnormal temporal summation [7]. For example, trigeminal neuralgia, which is caused by harmless stimulation, sudden onset, and termination, is characterized by electric shock or shooting pain and repeated attacks; chronic painful radiculopathy is persistent or recurrent pain caused by lesions or diseases involving the cervical, thoracic, lumbar, or sacral nerve roots; pain may be spontaneous but is usually aggravated or aroused by taking or maintaining a certain body posture or during exercise. The neuralgia caused by various central nervous system injuries is characterized by an enhanced response to painful stimuli (hyperalgesia) or a painful response to normally nonpainful stimuli (allodynia) [11].

Since NP is essentially a subjective experience described by patients' specific symptoms, current screening tools can only be expressed in the form of questionnaires, such as the neuropathic pain questionnaire, PainDetect, ID-Pain, and DN4, which classify NP according to the oral description of the pain quality reported by patients [12]. At the same time, the diagnosis of suspected NP requires a special examination to determine whether the pain originates from the nervous system. The distribution of pain must correspond to potential damage or diseases of the somatosensory nervous system [11]. Electrophysiological techniques and nerve biopsy samples can help assess the decline of neurological function and record the degree of neuropathy. However, noninvasive diagnostic techniques still need to be explored. In 2015, Tatullo et al. used bioelectrical impedance to detect oral lichen planus as a model of precancerous lesions. Compared with ordinary surgical biopsies, this method can be easily used in clinical practice and reduce patients' anxiety [13]. We look forward to the development of more nonoperative diagnostic techniques for the exact detection of NP in the future.

## 3. Current Therapeutic Strategies of Neuropathic Pain

Although NP is common, people with chronic pain usually do not get sufficient pain relief from current drugs. At present, first-line drugs for the treatment of NP are gabapentinoids (gabapentin and pregabalin), tricyclic antidepressants (TCAS), and selective serotonin-norepinephrine reuptake inhibitor (SNRI). Lidocaine, capsaicin, and tramadol have been recommended as second-line treatments, while strong opioids (morphine and oxycodone) and botulinum toxin A (BTX-A) are listed as third-line treatments for peripheral NP [14]. However, although calcium channel-activated anticonvulsants pregabalin and gabapentin, tricyclic antidepressants, and serotonin-norepinephrine reuptake inhibitors (duloxetine and venlafaxine) are used as first-line drugs, at present, only mild effects can be achieved in the clinical setting [15]. In order to improve compliance, it is necessary to explain to patients that these drugs are mainly used as painkillers, not for the treatment of mental disorders or epilepsy, and that because all drugs are central, they usually produce typical central side effects, such as sedation and dizziness; tricyclic antidepressants also have significant anticholinergic and sedative side effects as well as potential risks of falls. Topical use of drugs such as lidocaine, capsaicin 8% patches, and botulinum toxin A only had a short-term effect on patients with peripheral localized NP [7].

Treatment of NP remains a challenge. A major issue is that its etiology varies greatly and its mechanism is complex, including the peripheral, central, supraspinal, and central disinhibition mechanisms. We summarize the brief mechanisms of NP in Table 1. At present, the treatment of NP remains under continuous exploration and optimization in hopes of the emergence of novel effective drugs.

## 4. Development of Stem Cell Therapy

In recent years, stem cell therapy has shown sufficient promise to warrant a major position in the field of translational medicine. At present, a number of studies on MSCs used as therapeutic aids in clinical and surgical applications have been reported, such as MSC treatment for intervertebral disc regeneration and cell therapy as a promising auxiliary means for the cerebrovascular system [16]. Effective acquisition of stem cells has become an obstacle for practical application. Collecting bone marrow mesenchymal stem cells from human bone marrow (HBM) is not a simple process. In fact, donors must undergo invasive intervention for bone marrow to be extracted from the ilium. Isolated cells are not abundant because the frequency of bone marrow mesenchymal stem cells in bone marrow is low [17]. In 2013, Marrelli et al. demonstrated for the first time the presence of resident cells in periapical inflammatory tissue typical of MSCs: human Periapical Cyst-Mesenchymal Stem Cells (hPCy-MSCs) [18]. This new type of stem cell is located in the inner layer of the periapical inflammatory sac wall and is characterized by easy isolation from discarded tissues, which are often considered biological “waste.” hPCy-MSCs have extensive proliferation ability and the potential to differentiate into many cell types, such as adipocytes, osteoblasts, and neurons. Therefore, hPCy-MSCs can be regarded as an innovative source of stem cells for therapy [19]. It is worth noting that recent studies have shown that, in addition to biochemical factors, mechanical factors are increasingly considered as key regulatory factors in the behavior and function of dental pulp stem cells (DPSCs). A variety of mechanical stimuli can promote the proliferation and differentiation of MSCs. Low-intensity pulsed ultrasound (LIPUS) is considered to be one of the most promising mechanical stimuli for future clinical applications due to its economy, relative directness, and safety [20]. The increase of stem cell sources and the favorable effects of biochemical and mechanical factors on the proliferation and differentiation of MSCs provide more valuable insights for the development of stem cell-based therapy.

In terms of neuralgia, initially, researchers investigated the ability of stem cells to replace damaged nerve cells and transport nutritional factors to lesion sites; however, more recent research has shown that the effectiveness of stem cells against NP is mainly related to the two-way interaction between stem cells and resident cells in the damaged microenvironment [21]. Stem cells have the potential to block degeneration processes, inhibit apoptosis, and enhance survival/recovery in injured and uninjured nerves. Stem cells play nerve repair roles in both the central nervous system (CNS) and the peripheral nervous system (PNS). They can release a large number of neurotrophic factors, including epidermal growth factor, BDNF, NT-3, CNTF, basic fibroblast growth factor (bFGF/FGF-2), hepatocyte growth factor, and vascular endothelial growth factor (VEGF) [22, 23]. At present, a variety of stem cells including bone marrow mesenchymal stem cells (BMSCs), human amniotic fluid-derived mesenchymal stem cells (hAFMSCs), adipose-derived stem cells (ADSCs), and GABAergic intermediate neuron progenitor cells have strong therapeutic potential in the treatment of NP and the results are promising.

## 5. The Role of Stem Cells in the Peripheral Mechanism

### 5.1. Anti-Inflammatory Regulation

Peripheral sensitization plays an important role in the occurrence of NP symptoms after nerve injury. The accumulation of infiltrating immune cells such as neutrophils, macrophages, and mast cells at the site of nerve injury constitutes the peripheral cellular mechanism of overexcitation and continuous discharge of nerve fibers in neuropathy cases [24]. Inflammation releases a large number of chemical mediators, such as cytokines, chemokines, and lipid mediators, which sensitize and stimulate nociceptors and cause changes in the local chemical environment [25]. In animal models, there is sufficient evidence that anti-inflammatory cytokines have analgesic effects [26].

Stem cells have strong immunosuppressive and anti-inflammatory effects. By regulating and secreting various immunomodulatory factors, angiogenic factors, and nutritional factors, stem cells can reduce harmful immune responses and inflammation and repair different tissue injuries in different microenvironments [27, 28]. Studies have shown that stem cells can treat a variety of diseases such as heart failure and pulmonary fibrosis based on their anti-inflammatory effects [29, 30]. Presently, a number of NP-oriented stem cell studies attach importance to the anti-inflammatory effects, as shown in Table 2. In the study by Mert et al., adipose stem cell therapy significantly decreased the levels of proinflammatory factors such as IL-1 *β* and IL-6 induced by the chronic constriction nerve injury model (CCI) in the sciatic nerve and increased anti-inflammatory factor IL-10 [31]. This may be the result of the interaction between stem cells and monocytes/macrophages, as stem cells promote the polarization of macrophages to anti-inflammatory phenotypes. To demonstrate that the anti-inflammatory and analgesic effects of stem cells are mediated by monocytes/macrophages, Guo et al. used a liposome-encapsulated chlorophosphonate method (Lipo-CLO) to deplete monocytes/macrophages; they found that Lipo-CLO treatment reduced the analgesic effects produced by BMSCs. The peripheral blood mononuclear cells of rats that were treated with BMSCs were isolated. The results showed that the expression of some markers of M2 macrophages increased after BMSC treatment, while the expression of genes related to M1 macrophages decreased, suggesting that BMSCs promoted the polarization of macrophages to anti-inflammatory phenotype [32]. Similarly, Omi et al. demonstrated that dental pulp stem cell (DPSC) transplantation increased the M2 phenotype of sciatic nerve macrophages in diabetic rats, and DPSC-conditioned medium promoted M2 macrophage marker gene expression of RAW264.7 cells in vitro [33].

**Figure 1:**
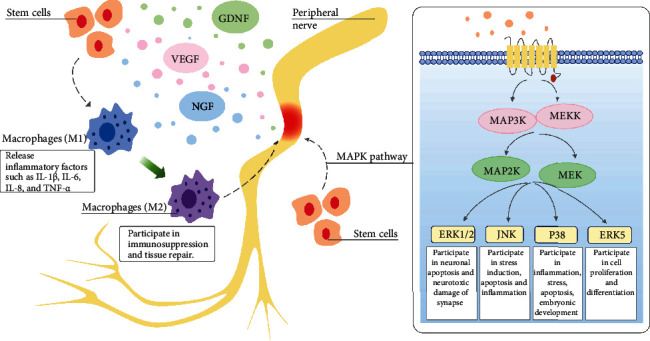
Diagram showing the role of stem cells in peripheral nerve injury and the simplified MAPK pathway. GDNF = glial-derived neurotrophic factor; IL = interleukin; NGF = nerve growth factor; TNF = tumor necrosis factor; VEGF = vascular endothelial growth factor.

As shown in Figure 1, stem cells also play an anti-inflammatory role through the mitogen-activated protein kinase (MAPK) pathway. After nerve injury, signals from damaged axons lead to the activation of the extracellular signal-related MAPK signal pathway in Schwann cells; this is one of the earliest events to trigger the expression of inflammatory mediators and recruit immune cells to the injured nerve [25]. There are four subsets of the MAPK pathway, among which ERK1/2 and P38 play key roles in the induction and maintenance of chronic pain. In the rat CCI model, intrathecal injection of BMSCs showed that stem cells inhibited the expression of pERK1/2 in dorsal root ganglion (DRG) induced by CCI [34]. Almassri et al. achieved the same results in the treatment of paclitaxel- (PTX-) induced peripheral neuropathy with BMSCs. BM-derived mesenchymal stem cells (MSCs) reversed the increased expression of p-p38-MAPK protein induced by PTX and decreased the expression of inflammatory factors such as NF-*κ*B p65, TNF-*α*, and IL-6 [35]. Stem cells can have dynamic anti-inflammatory effects in many aspects, which is an advantage and characteristic of cell therapy compared to monotherapy.

### 5.2. Neuroprotection and Promotion of Axonal Myelin Regeneration

Nerve injury causes abnormal neuron excitability, induces nerve fiber degeneration, and changes channel expression and composition, resulting in ectopic discharges. Spontaneous ectopic activity on nerve endings or axons is important for spontaneous pain and is a driving factor for abnormal pain response [24, 36, 37]. Activating transcription factor 3 (ATF3) is a widely used marker of DRG neuronal injury. Chen et al. found that the immunoreactivity (IR) of ATF3 in L4-L5 DRG neurons significantly increased by 40% in the CCI model. Four days after intrathecal BMSC injection, ATF3 expression in DRG neurons induced by CCI was inhibited by 14%. Nerve injury can also downregulate the neuropeptide calcitonin gene-related peptide (CGRP) in peptidergic neurons and the isolectinB4 (IB4) bound by nonpeptidergic neurons in DRGs. BMSCs reversed the downregulation of CGRP and IB4 in DRG neurons induced by CCI and protected DRG neurons from axonal injury [38]. Chiang et al. also observed that human AFMSCs reversed the downregulation of nerve injury marker protein gene product 9.5 (PGP9.5) and S100 calcium-binding protein induced by CCI in the treatment of CCI with hAFMSCs [39]. These results directly demonstrate that stem cell therapy reduces persistent nerve damage.

Glial-derived neurotrophic factor (GDNF) has long been shown to be a growth factor that can successfully reverse and normalize NP in rats and play a neuroprotective role. Many studies have used GDNF and its receptor as hot spots in the development of new painkillers [40, 41]. Sarveazad et al. studied the treatment of spinal cord injury with human ADSCs; Bielschowsky's staining showed that hADSC treatment increased the number of axons around the cavity formed by spinal cord injury. Additionally, GDNF mRNA expression increased after hADSC transplantation. Stem cells may increase the survival of motor and sensory neurons, improve motor function, induce neurogenesis and axon growth, enhance myelin formation, and relieve pain by regulating GDNF [42]. Similarly, the use of genetically engineered neural stem cells specifically expressing enhanced green fluorescent protein (for localization) and GDNF in the treatment of spinal cord nerve ligation in spinal nerve ligation (SNL) rats can achieve a more significant effect [43]. In addition to GNDF, Al-Massri et al. also found that stem cells can reverse the decrease of nerve growth factor (NGF) in patients with nerve injury and maintain the neuroprotective effect of NGF by promoting axonal growth and neuronal maintenance and survival [35].

Additionally, VEGF is an important regulator of nerve regeneration, which can support and promote the growth of regenerated nerve fibers through the combination of angiogenesis, neuronutrition, and neuroprotection, so as to restore nerve function [44–46]. Lee et al. demonstrated that transplantation of neural stem cells expressing VEGF increased functional recovery, pain relief, myelin formation, and vascular count in sciatic nerve injury model rats [47]. However, Di Cesare Mannelli and colleagues arrived at a different conclusion. In their experiment, the concentration of VEGF in the spinal ganglion and spinal cord increased in oxaliplatin-induced neuralgia in rats but significantly decreased after administration of ADMSCs [48]. The different results may be attributed to the balance of VEGF isoforms. VEGF-A165a enhances the sensitivity of peripheral nociceptive neurons by acting on VEGFR2 and TRPV1-dependent mechanisms, thus enhancing nociceptive signal transduction. VEGF-A165b can block the effect of VEGF-A165a. Blocking the proximal splicing event—leading to the preferential expression of VEGF-A165b over VEGF165a—prevents pain in vivo [49]. Stem cell therapy plays a uniquely balancing role in VEGF regulation; however, the specific interaction between VEGF and stem cells needs more exploration.

The different results may be due to the different pain models and stem cell microenvironments. Since it induces angiogenesis, VEGF participates in tumor-related pain in mouse models of cancer-related pain (such as osteolytic sarcoma, implanted breast cancer of the femur, lung cancer, and pancreatic cancer) [50]. The mechanism of pain caused by the increase of VEGF caused by oxaliplatin is similar to that of cancerous neuralgia. Stem cell therapy plays a uniquely balancing role in different microenvironments; however, the specific interaction between VEGF and stem cells needs more exploration.

## 6. The Role of Stem Cells in the Spinal Mechanism

### 6.1. Weakening and Reversing Central Sensitization

**Figure 2:**
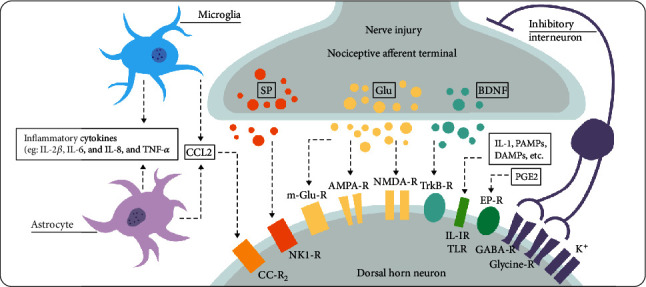
Diagram showing the synaptic junction in the dorsal horn of the spinal cord. Reprinted with permission from Cohen and Mao [54]. Copyright © 2020, British Medical Journal Publishing Group. AMPA = *α*-amino-3-hydroxy-5-methyl-4-isoxazolepropionic acid; BDNF = brain-derived neurotrophic factor; CCL = chemokine (C-C motif) ligand; CC-R2 = CC-chemokine receptor; DAMPs = danger-associated molecular patterns; EPR = prostaglandin E2 sensitive receptor; GABA = *γ*-aminobutyric acid; Glu = glutamate; IL = interleukin; m-Glu = metabotropic glutamate; NK = neurokinin; NMDA = N-methyl-D-aspartate; PAMPs = pathogen-associated molecular patterns; PG = prostaglandin; -R = receptor; SP = substance P; TLR = toll-like receptor; TNF = tumor necrosis factor; Trk = tyrosine kinase.

Central sensitization, characterized by increased neuronal excitability, is considered to be one of the most important mechanisms leading to NP.  Figure 2 shows the synaptic connections in the dorsal horn of the spinal cord; the glutamate receptor is indispensable for central sensitization development. After nerve injury, the release of excitatory amino acid (glutamate) in the spinal dorsal horn is enhanced and the excitatory N-methyl-d-aspartate (NMDA) receptor (NMDAR) is continuously activated to maintain the afferent nerve transmission to the sensory brain [51–53]. Under the long-term stimulation of chronic nerve injury, NMDAR is upregulated, thus establishing a state of central sensitization [51]. Many animal models have shown that blocking NMDAR can relieve NP [54]. Specific antagonists of NMDARs have been used intermittently for NP [55]. Guo et al. intravenously injected BMSCs into tendon ligation (TL) and SNL rat models. They found that BMSCs could inhibit the expression of NMDA receptors and protect them from glutamate excitotoxicity, which alleviated the mechanical hyperalgesia after spinal cord injury in rats and demonstrated the beneficial analgesic properties of stem cells to chronic pain [56].

Studies have shown that transforming growth factor-*β*1 (TGF-*β*1) attenuates glutamate-induced excitotoxic neuronal damage in rat neocortical neurons in a concentration-dependent manner [57]. TGF-*β*1 regulates excitatory synaptic transmission of spinal cord neurons after chronic brain injury through the TGF-*β* receptor 1. Chen et al. found that the expression of TGF-*β*1 in cerebrospinal fluid increased when BMSCs were used to treat neuralgia in mice. They found that the basal release of TGF-*β*1 from the culture medium of BMSCs was very high. To determine whether TGF-*β*1 was involved in the antinociceptive effect of BMSCs in NP, mice were treated with a specific neutralizing antibody against TGF mRNA 3 days after BMSC injection. Subsequently, the experimental results showed that neutralization of TGF-*β*1 expression reversed the antihyperalgesia effect of BMSCs [38]. Thus, the data show that stem cells can reduce the increase of neuronal excitability after nerve injury by releasing TGF-*β*1, resist central sensitization, and thus exert an analgesic effect.

### 6.2. Inhibition of Glial Cell Activation

Many studies have demonstrated that the long-term analgesic and therapeutic effects of stem cells are closely related to the role of glial cells (Table 3). Glial cells account for approximately 70% of the central nervous system cells and play an important role in maintaining balance in the body [54]. Glial cells are divided into three types: astrocytes, oligodendrocytes, and microglia [58]. The literature shows that microglia are activated within 24 hours after nerve injury; astrocytes are activated soon after nerve injury and the activation lasts for 12 weeks [59]. The subsequent release of cytokines from astrocytes and microglia induces a series of cellular responses, such as upregulation of glucocorticoid and glutamate receptors, leading to spinal cord excitation and neuroplasticity. This is closely related to the symptoms of NP, such as pain hypersensitivity [54, 59].

Stem cells can effectively inhibit the activation of glial cells. For example, the expression of GFAP (astrocyte marker) in the spinal dorsal horn of CCI rats is elevated. Intravenous administration of ADSC lowers the expression of GFAP to 1.2 times that of the control group or close to the control group [60]. Intrathecal injection of BMSCs can downregulate microglial activity in the ipsilateral and contralateral spinal cord dorsal horns of rats with noncompressive disc herniation and mediate the behavioral hypersensitivity related to nerve root pain by reducing the production of inflammatory cytokines produced by activated spinal microglia [61]. Romero-Ramirez et al. stained microglia with anti-Iba1 antibodies and found that after spinal cord injury, the expression of Iba1 in the lesion center was 10 times stronger than that in rats without spinal cord injury. However, Iba1 expression only increased 4 times in animals implanted with BMSC, suggesting that injected cells decreased the activation of microglia [62]. The MAPK signal pathway is activated after microglial activation, which promotes long-term potentiation and central sensitization in pain. Stem cells effectively inhibit microglial activation and also inhibit the MAPK signal pathway activation in activated glial cells. The MAPK signal cascade is indicated by phosphorylation, which activates ERK1/2, JNK, and p38MAPK, which in turn leads to the phosphorylation and activation of transcription factor CREB, which affects pain development through NMDA [63]. BMSC decreased the activation of p-p38MAPK and p-ERK1/2 in microglia induced by spinal cord injury (SCI), and the expression of CREB and PKC-c in injured and surrounding dorsal horn neurons, alleviated SCI-induced neuralgia, and improved motor function in rats [64].

Stem cells are also involved in the interaction between glial cells. After nerve injury, astrocytes can increase the expression of chemokine CCL7, which is a common activator of microglia in the spinal cord under the condition of NP. Li et al. used BMSCs pretreated with IL-1*β* to treat the SNL rat model. The inhibitory effect of IL-1*β*-BMSCs on microglial activation and NP was mediated by reduced CCL7 in the spinal cord; promoting astrocyte activation could alleviate the inhibitory effect of IL-1 *β*-BMSC-mediated downregulation of CCL7. It is speculated that stem cells themselves not only inhibit the activation of microglia and astrocytes but also reduce the activation of microglia by inhibiting astrocytes' secretion of CCL7 [65].

However, some experiments have suggested different conclusions. Teng et al. believe that intrathecal BMSCs alleviate NP through microglial activity independent of microglial activation. In their experiment, stem cells inhibited the core pain signal pathway of P2X4R in microglia and reduced the expression of P2X4R. However, it was found that the number of activated microglia was not affected by IBA labeling of microglia [66]. Compared to the previous three-week studies, the results from Teng et al. were taken from the chronic compression of the dorsal root ganglion model (CCD) six days after stem cell therapy. Therefore, different treatment outcomes may be related to different treatment durations. The mechanism of stem cell and glial cell interactions on pain needs to be explored in more detail.

### 6.3. Reduced Apoptosis and Autophagy of Spinal Cord Cells

As mentioned in the peripheral mechanism, the control of nerve injury is an important part of preventing the development of NP. Stem cells not only promote the recovery of peripheral nerve injury but also play the same role in the central nervous system. Lin et al. found that elevated TUNEL expression, a marker of apoptosis in the spinal cord, was reversed when ASCs were subcutaneously transplanted as treatment in the burn rat model of NP. Additionally, there was a significant reduction in LC3B-II and Beclin1 in the spinal dorsal horn cells, which was related to inflammation and apoptosis [67]. Experiments by Sarveazad et al. and Romero-Ramirez revealed that stem cell therapy increased the number of axons around the cavity and reduced the size of the cavity after spinal cord injury [42, 62]. Stem cells reduce spinal cord apoptosis and promote the recovery of injured nerves, which play an important role in the analgesia and treatment of NP. The general contents of the experimental studies related to the spinal mechanism are shown in Table 3.

## 7. Transplantation of Stem Cells after Differentiation *In Vitro* Reduces Disinhibition at the Spinal Cord Level

Peripheral and central nervous system injuries are often the leading cause of chronic NP. In the spinal cord, local intermediate neurons and descending inhibitory circuits regulate pain sensation in the superficial layer of the spinal dorsal horn [68]. The GABA pathway plays an important role in the regulation of the balance between excitability and inhibition in synaptic transmission. GABA (*γ*-aminobutyric acid) is a widely distributed inhibitory neurotransmitter in the spinal cord, which balances the enhancement of synaptic transmission in neurons after spinal cord injury mediated by glutamate [69]. As shown in Figure 2, the activation of intermediate inhibitory neurons leads to the release of neurotransmitter GABA, which inhibits postsynaptic neurons through membrane hyperpolarization [70]. Drugs that block the transmission of GABA nerves or the loss of specific subunits of GABA receptors in the spinal cord can lead to hyperalgesia and hypersensitivity [71]. After spinal cord injury, the function of GABA in the spinal dorsal horn decreases, and the loss of inhibitory intermediate neurons leads to overexcitation of spinal cord neurons and an increase of neuronal sensitivity, which leads to chronic NP [71–74].

However, systemic application of GABA enhancers cannot effectively relieve NP and they have significant adverse reactions. Therefore, the idea of directly transplanting GABA secretory cells or GABA neurons into the spinal cord has aroused considerable interest in NP [75, 76]. Based on the fact that the transplantation of GABAergic intermediate neuron progenitor cells can reduce neuronal overexcitability, Fandel et al. performed a study using human embryonic stem cells (HESCs). First, they induced HESCs into medial ganglion eminence- (MGE-) like cells (HESC-MGEs). Two weeks after thoracic spinal cord injury in mice, the hESC-MGEs were transplanted into the lumbar spinal cord. The transplanted hESC-MGEs migrated to the injured site and differentiated into subtypes of GABA neurons, forming synaptic connections in the local loop to reduce CNP caused by spinal cord injury [77]. Similarly, Hwang et al. induced mouse embryonic stem cells (MESCs) to differentiate into spinal cord GABA neurons *in vitro* and transplanted them into the spinal cord of model rats 21 days after spinal cord injury. The changes of mechanical hypersensitivity in rats before and after transplantation were observed; spinal cord implanted GABA neurons had evident NP-relieving effects [78].

Additionally, Tashiro et al. transplanted neural stem/progenitor cells into the spinal cord of SCI mice to reduce pain in model mice; they also found an increase in GABA activity in the dorsal horn of the spinal cord [79]. Although this study did not use neural stem cells that have differentiated into GABAergic neurons, the results showed that NSCs continued to be involved in the GABAergic pathway. More studies are needed to explore this specific mechanism.

## 8. Stem Cells Can Accumulate at the Site of Nerve Injury through the CXCL12-CXCR4 Axis

Stem cells have the ability to homing, that is, they can migrate to damaged/repaired areas, which determines their effectiveness in cell therapy [80]. The trafficking of MSCs from their niche to target tissues is a complex process. This delivery process is affected by both chemical factors (such as chemokines, cytokines, and growth factors) and mechanical factors (such as hemodynamic forces applied to the vessel walls in the forms of shear stress, vascular cyclic stretching, and extracellular matrix (ECM) stiffness) [81]. At present, the research on the homing of stem cell therapy for NP is mainly focused on the CXCL12-CXCR4 axis. Chemokines are low-molecular-weight proteins, which can promote the migration and adhesion of their target cells. Functionally, chemokines can be divided into inflammatory or steady-state chemokines according to their induced or structural products [82]. Inflammatory chemokines are induced by inflammatory stimulation to attract leukocytes from circulation towards the sites of infection or injury, while steady-state chemokines are structurally expressed and regulate cell transport and homing during development and immune surveillance [83, 84]. Chemokine stromal cell-derived factor-1 (SDF-1)/CXCL12 is such a dynamically balanced CXC chemokine and is a single natural ligand of chemokine receptor CXCR4 [82].

Data show that the CXCL12-CXCR4 chemokine receptor axis plays an important role in embryonic cell line homing [85]. In NP, nerve and spinal cord injury is often accompanied by an increase in CXCL12. Experimental studies have confirmed that the animal model of spared nerve injury (SNI) increases the expression of CXCL12 and its homologous receptor CXCR4 in neurons and satellite glial cells of lumbar 5 DRG. SNI also induced sustained upregulation of CXCL12 and CXCR4 expression in ipsilateral L4-5 spinal dorsal horn [86]. In the SNL model, the CXCL12/CXCR4 signaling pathway is involved in the occurrence and maintenance of NP through the central sensitization mechanism [87]. The transplanted stem cells can express CXCR4 receptors, and some studies have confirmed that CXCL12 can promote the migration of stem cells *in vitro* [88, 89]. The increase in CXCL12 caused by NP can promote the migration of CXCR4-expressing stem cells in the body, which is supported by animal experiments *in vivo*. Berta et al. used the intrathecal injection of BMSCs to reveal their role and analgesic effect in NP caused by nerve injury: most of the injected BMSCs were detected around the injured DRG tissue. BMSCs are selectively recruited into the DRG tissue of damaged neurons through the CXCL12/CXCR4 axis; they survived for a long time in the tissue and played a continuous analgesic effect [90]. Chen et al. used CXCR4 siRNA to reduce CXCR4 mRNA levels by 85%. After intrathecal injection of siRNA-treated BMSCs, the number of stem cells that migrated to the injured dorsal root ganglion was significantly reduced, and the inhibitory effect on NP mechanical hypersensitivity was also weakened [38]. Some studies have suggested that intravenous injection of MSCs can trap them in the lungs, but experimental evidence shows that MSCs can home to damaged tissue after systemic delivery [91, 92]. In the CCI rat model, inflammation guided the transplantation of MSCs to migrate towards the injured site. Other experimental results showed that MSCs reaching the injured site were recruited by CXCL12 (SDF-1, 39). Thus, the CXCL12-CXCR4 axis plays an important role in the homing of stem cells in NP.

## 9. Current Clinical Research Progress and Challenges Faced by Stem Cells

The surprising results from a large number of stem cell preclinical trials in the treatment of NP have prompted scientists to focus on the corresponding clinical trials. In 2014, Mendonça's team conducted a phase I uncontrolled study of 14 patients with chronic traumatic spinal cord injury [93]. They cultured autologous BMSCs *in vitro* and transplanted them directly into the patient's spinal cord injury site. The clinical pain symptoms of the subjects improved by varying degrees, and only one patient developed cerebrospinal fluid leakage due to postoperative complications caused by the surgical procedure, which had nothing to do with the stem cells themselves [93]. Vickers et al. used autologous adipose MSCs to treat 10 patients with neurotrigeminal neuralgia. This stem cell therapy had no systemic or local tissue side effects; after 6 months, the pain intensity score and the use of antineurotic drugs were decreased in 5/9 subjects [94]. In 2018, the Vaquero's team put forward guidelines for the treatment of spinal cord injury by intrathecal injection of autologous spinal cord MSCs (three doses of 100 million MSCs were given at intervals of 3 months) and explored the safety and effectiveness of the guidelines [95]. In clinical trials of 10 patients with chronic spinal cord injury, the results showed that the intensity of NP was significantly and gradually improved after the first BMSC injection, and autologous BMSCs were safely tolerated [95, 96].

Although preliminary clinical trials have yielded good results, there are still many challenges in stem cell therapy for NP. First, direct intramedullary transplantation or intrathecal injection is often used in the treatment of NP related to spinal cord injury. The invasive surgical process brings more risks to the treatment, and the safety and tolerance of cell injection in different segments are also very different [97]. NP patients may not be willing to take the extra risks. Although preclinical studies have shown that both intrathecal and intravenous injection can significantly reduce NP [98], this review also briefly describes the partial homing mechanism of stem cells, but the researchers are at a loss about the whole pathway of stem cells entering the systemic circulation. Second, autologous stem cells are used in preliminary clinical trials, which are obtained from patients themselves, so the risk of rejection is negligible [99]. We expect stem cells to become a therapeutic drug, and the use of allogeneic expansion of stem cells in the future is inevitable. However, challenges remain, such as solving possible immune rejection and reducing the cost of obtaining stem cells to make it easier for NP patients.

In early human trials, cell intervention requires a more comprehensive assessment to ensure risk levels are reasonable and based on solid evidence of preclinical validity [100]. Treatments that do not provide a clear mechanism or reasonable theoretical basis and lack preclinical evidence of effectiveness, proof of concept, or safety are unlikely to be ready for clinical trials [101]. Clearly, more preclinical studies are needed to elaborate the treatment and homing mechanisms in order to provide theoretical reference for clinical trials of stem cell therapy for NP in the future.

## 10. Conclusion

The mechanism of NP is extremely complex, and it is difficult to achieve good results by using current clinical first-line drugs. Owing to the two-way interaction between stem cells and resident cells in the damaged microenvironment, stem cells can play multiple roles, such as peripheral, central, and spinal cord disinhibition, which significantly reduces the occurrence of clinical symptoms including spontaneous pain, hyperalgesia, and hyperalgesia. We look forward to the summary and analysis of the mechanisms related to the treatment of NP by stem cells, which can provide theoretical reference for preclinical and clinical research in the future and contribute to the field of stem cell therapy and pain.

## Figures and Tables

**Table 1:** Mechanisms of neuropathic pain.

Peripheral mechanisms Peripheral sensitization Cascade release of inflammatory mediators and nociceptive sensitivity Dorsal root ganglion and damaged nerve fibers produce ectopic discharges Expression of ion channels Multiple sodium and calcium channels' expression was increased/decreased and the stimulation threshold decreased Phenotypic switch The phenotype of nerve fibers changed and the neuromodulator of C fibers increased Sensory denervation and the sprouting of collateral nerve fibers Sympathetic maintained pain Sensory neurons were sensitized and release of norepinephrine increased
Spinal mechanisms Central sensitization Bone marrow excitatory glutamate receptor is activated, which increases the excitability of neurons, and C fibers are repeatedly activated Glial cell activation Activation of glial cells increased the release of proinflammatory factors
Supraspinal mechanisms Pain signal transduction changes Neurotransmitter metabolism changes
Central disinhibition Restrain current loss Apoptosis of inhibitory intermediate neurons in the spinal cord Regulation of descending inhibition

**Table 2:** Preclinical study of stem cells involved in peripheral mechanism in the treatment of neuropathic pain.

Cell type (source)	Delivery site	Cell number	Model of NP and species	Brief peripheral mechanism	Author and year
ADMSCs (rats)	i.p. Local application	2 × 10^6^ 1 × 10^6^	CCI (rats)	Decreased IL-1*β* and IL-6 in the sciatic nerve and increased IL-10 expression.	Mert et al. [31]
AM1241-pretreated BMSCs (SCB)	i.t.	2 × 10^5^	CCI (mice)	Inhibited CCI-induced p-ERK1/2 expression in the DRGs, and increased the amount of TGF-*β*1 protein in the DRGs. TGF-*β*1 attenuated NP through inhibition of p-ERK1/2.	Xie et al. [34]
BMSCs (rats)	i.v.	1 × 10^6^	Paclitaxel-induced neuropathy (rats)	Increased expression of NGF in the sciatic nerve, reversed the increase of NF-*κ*Bp65, TNF-*α*, and IL-6 caused by CCI.	Al-Massri et al. [35]
BMSCs (mice)	i.t.	1.5 × 10^5^/2.5 × 10^5^	CCI (mice)	Inhibited expression of ATF3 in DRG neurons induced by CCI. Reversed the downregulation of CGRP and IB4 staining in central axon terminals of DRG neurons and spinal dorsal horn caused by CCI.	Chen et al. [38]
AFMSCs (human)	i.v.	5 × 10^5^ × 3d	CCI (rats)	Increased the expression of IL-1 *β*, CD68, and TNF-*α*, and decreased the expression of S100 and neurofilament in the injured nerve.	Chiang et al. [39]
ADSCs (human)	Direct implantation of the injured site	1 × 10^6^	SCI (rats)	Increased the transcription of GDNF and decreased the expression of IL-6 at the injured site.	Sarveazad et al. [42]
SV-VEGF-NSCs (ATCC)	Direct implantation of the injured site	1 × 10^5^	Sciatic nerve crush injury (rats)	The expression of VEGF could increase cell viability, promote myelin regeneration, and sciatic nerve angiogenesis.	Lee et al. [47]
ADSCs (rats)	i.v.	2 × 10^6^	Oxaliplatin-induced neuropathy (rats)	Reversed the increase of VEGF concentration induced by oxaliplatin.	Di Cesare Mannelli et al. [48]

Notes: the above-mentioned experimental studies have shown that the stem cells used in the study are effective and analgesic in the treatment of neuropathic pain in this model. Abbreviations: CCI: chronic constriction nerve injury model; SCI: spinal cord injury model; BMSCs: bone marrow mesenchymal stem cells; ADSCs: adipose-derived stem cells; AFMSCs: amniotic fluid-derived mesenchymal stem cells; SV-VEGF-NSCs: vascular endothelial growth factor-expressing neural stem cell; i.p.: intraperitoneal injection; i.v.: intraperitoneal injection; i.t.: intrathecal injection; SCB: Stem Cell Bank (Chinese Academy of Sciences); ATCC: American Type Culture Collection (CRL-2925; ATCC, Manassas, Virginia, USA).

**Table 3:** Preclinical study of stem cells involved in spinal mechanism in the treatment of neuropathic pain.

Cell type (source)	Delivery site	Cell number	Model of NP and species	Brief spinal mechanism	Author and year
BMSC (rats)	i.v.	1 × 10^6^	SNL (rats)	Inhibited the phosphorylation of GluN2A in RVM, reduced the expression of PKCG, inhibited the expression of NMDA receptors, thus resisting the development of central sensitization.	Guo et al. [56]
BMSCs (mice)	i.t.	1.5 × 10^5^/2.5 × 10^5^	CCI (mice)	Released TGF-*β*, regulated the excitatory synaptic transmission of spinal cord neurons, and reduced the increase in neuronal excitability after nerve injury, thus resisting the development of central sensitization.	Chen et al. [38]
BMSCs (rats)	i.v.	1 × 10^6^	CCI (rats)	Decreased the increase of GFAP expression in rat spinal cord induced by CCI, reduced the expression of TGF-*α*, and reduced the apoptosis of tissue cells.	Forouzanfar et al. [60]
BMSCs (rats)	i.t.	1 × 10^6^	Noncompressive disk herniation (rats)	Decreased the mRNA and protein expression of TNF-*α* and IL-1*β*, upregulated the expression of TGF-*β*, and reduced the activation of microglia in the dorsal horn of the spinal cord.	Huang et al. [61]
BMSCs (human)	i.t.	2.3 ± 0.5 × 10^6^	SCI (rats)	Reduced the activation of spinal microglia, apoptosis, and autophagy of spinal cord cells.	Romero-Ramirez et al. [62]
BMSCs (mice)	Direct implantation of the injured site	2 × 10^5^	SCI (mice)	Decreased the activation of p-p38MAPK and pERK1/2 in microglia induced by SCI, and the expression of CREB and PKC-c in injured and surrounding dorsal horn neurons.	Watanabe et al. [64]
IL-1*β*-BMSCs (rats)	i.t.	2.5 × 10^6^	SNL (rats)	Decreased the activation of astrocytes in the spinal cord and reduced the expression level of CCL7 in the spinal cord, thus inhibiting the activation of microglia.	Li et al. [65]
BMSCs (rats)	i.t.	1 × 10^6^	CCD (rats)	Inhibited the expression of P2X4R in spinal microglia but did not affect the activation of microglia induced by CCD.	Teng et al. [66]
ADSCs (autologous, rats)	s.c.	1 × 10^6^	Burn-induced neuropathic pain (rats)	Reduced the expression of astrocytes in the spinal cord and reduced the apoptosis and autophagy of spinal cord cells.	Lin et al. [67]
ADSCs (human)	Direct implantation of the injured site	1 × 10^6^	SCI (rats)	Reduced the syringomyelia caused by SCI and increased the number of axons around the cavity.	Sarveazad et al. [42]

Notes: the above-mentioned experimental studies have shown that the stem cells used in the study are effective and analgesic in the treatment of neuropathic pain in this model. Abbreviations: SNL: spinal cord nerve ligation model; CCI: chronic constriction nerve injury model; SCI: spinal cord injury model; CCD: chronic compression of the dorsal root ganglion model; BMSCs: bone marrow mesenchymal stem cells; IL-1*β*-BMSCs: interleukin-1*β* pretreated bone marrow stromal cells; ADSCs: adipose-derived stem cells; i.v.: intraperitoneal injection; i.t.: intrathecal injection; s.c.: subcutaneous injection.

## Data Availability

Previously reported data were used to support this study and are available at DOI. These prior studies (and datasets) are cited at relevant places within the text as references.

## References

[1] Inoue K., Tsuda M. (2018). Microglia in neuropathic pain: cellular and molecular mechanisms and therapeutic potential. *Nature Reviews. Neuroscience*.

[2] Treede R. D., Rief W., Barke A. (2019). Chronic pain as a symptom or a disease: the IASP classification of chronic pain for the International Classification of Diseases (ICD-11). *Pain*.

[3] Breivik H., Collett B., Ventafridda V., Cohen R., Gallacher D. (2006). Survey of chronic pain in Europe: prevalence, impact on daily life, and treatment. *European Journal of Pain*.

[4] Nahin R. L. (2015). Estimates of pain prevalence and severity in adults: United States, 2012. *The Journal of Pain*.

[5] Riskowski J. L. (2014). Associations of socioeconomic position and pain prevalence in the United States: findings from the National Health and Nutrition Examination Survey. *Pain Medicine*.

[6] Widerstrom-Noga E. (2017). Neuropathic pain and spinal cord injury: phenotypes and pharmacological management. *Drugs*.

[7] Gierthmuhlen J., Baron R. (2016). Neuropathic pain. *Seminars in Neurology*.

[8] Bouhassira D. (2019). Neuropathic pain: definition, assessment and epidemiology. *Revue Neurologique*.

[9] Treede R. D., Jensen T. S., Campbell J. N. (2008). Neuropathic pain: redefinition and a grading system for clinical and research purposes. *Neurology*.

[10] Jensen T. S., Baron R., Haanpää M. (2011). A new definition of neuropathic pain. *Pain*.

[11] Scholz J., Finnerup N. B., Attal N. (2019). The IASP classification of chronic pain for ICD-11: chronic neuropathic pain. *Pain*.

[12] Baron R., Binder A., Wasner G. (2010). Neuropathic pain: diagnosis, pathophysiological mechanisms, and treatment. *Lancet Neurology*.

[13] Tatullo M., Marrelli M., Amantea M. (2015). Bioimpedance detection of oral lichen planus used as preneoplastic model. *Journal of Cancer*.

[14] Cavalli E., Mammana S., Nicoletti F., Bramanti P., Mazzon E. (2019). The neuropathic pain: an overview of the current treatment and future therapeutic approaches. *International Journal of Immunopathology and Pharmacology*.

[15] Finnerup N. B., Attal N., Haroutounian S. (2015). Pharmacotherapy for neuropathic pain in adults: a systematic review and meta-analysis. *The Lancet Neurology*.

[16] Ballini A., Cantore S., Scacco S., Coletti D., Tatullo M. (2018). Mesenchymal stem cells as promoters, enhancers, and playmakers of the translational regenerative medicine 2018. *Stem Cells International*.

[17] Beyer Nardi N., da Silva Meirelles L. (2006). Mesenchymal stem cells: isolation, in vitro expansion and characterization. *Handbook of Experimental Pharmacology*.

[18] Marrelli M., Paduano F., Tatullo M. (2013). Cells isolated from human periapical cysts express mesenchymal stem cell-like properties. *International Journal of Biological Sciences*.

[19] Spagnuolo G., Codispoti B., Marrelli M., Rengo C., Rengo S., Tatullo M. (2018). Commitment of oral-derived stem cells in dental and maxillofacial applications. *Dentistry Journal*.

[20] Marrelli M., Codispoti B., Shelton R. M. (2018). Dental pulp stem cell mechanoresponsiveness: effects of mechanical stimuli on dental pulp stem cell behavior. *Frontiers in Physiology*.

[21] Franchi S., Castelli M., Amodeo G. (2014). Adult stem cell as new advanced therapy for experimental neuropathic pain treatment. *BioMed Research International*.

[22] Chen L., Huang H., Sharma H. S., Zuo H., Sanberg P. R. (2013). Cell transplantation as a pain therapy targets both analgesia and neural repair. *Cell Transplantation*.

[23] Fortino V. R., Pelaez D., Cheung H. S. (2013). Concise review: stem cell therapies for neuropathic pain. *Stem Cells Translational Medicine*.

[24] Meacham K., Shepherd A., Mohapatra D. P., Haroutounian S. (2017). Neuropathic pain: central vs. peripheral mechanisms. *Current Pain and Headache Reports*.

[25] Ellis A., Bennett D. L. H. (2013). Neuroinflammation and the generation of neuropathic pain. *British Journal of Anaesthesia*.

[26] Sommer C., Leinders M., Uceyler N. (2018). Inflammation in the pathophysiology of neuropathic pain. *Pain*.

[27] Harrell C. R., Jankovic M. G., Fellabaum C. (2019). Molecular mechanisms responsible for anti-inflammatory and immunosuppressive effects of mesenchymal stem cell-derived factors. *Advances in Experimental Medicine and Biology*.

[28] Takizawa N., Okubo N., Kamo M. (2017). Bone marrow-derived mesenchymal stem cells propagate immunosuppressive/anti-inflammatory macrophages in cell-to-cell contact-independent and -dependent manners under hypoxic culture. *Experimental Cell Research*.

[29] Epstein S. E., Lipinski M. J., Luger D. (2018). Persistent inflammation, stem cell-induced systemic anti-inflammatory effects, and need for repeated stem cell injections: critical concepts influencing optimal stem cell strategies for treating acute myocardial infarction and heart failure. *Journal of the American Heart Association*.

[30] Chen S., Cui G., Peng C. (2018). Transplantation of adipose-derived mesenchymal stem cells attenuates pulmonary fibrosis of silicosis via anti-inflammatory and anti-apoptosis effects in rats. *Stem Cell Research & Therapy*.

[31] Mert T., Kurt A. H., Altun I., Celik A., Baran F., Gunay I. (2017). Pulsed magnetic field enhances therapeutic efficiency of mesenchymal stem cells in chronic neuropathic pain model. *Bioelectromagnetics*.

[32] Guo W., Imai S., Yang J. L. (2017). In vivo immune interactions of multipotent stromal cells underlie their long-lasting pain-relieving effect. *Scientific Reports*.

[33] Omi M., Hata M., Nakamura N. (2016). Transplantation of dental pulp stem cells suppressed inflammation in sciatic nerves by promoting macrophage polarization towards anti-inflammation phenotypes and ameliorated diabetic polyneuropathy. *Journal of Diabetes Investigation*.

[34] Xie J., Ren J., Liu N. (2019). Pretreatment with AM1241 enhances the analgesic effect of intrathecally administrated mesenchymal stem cells. *Stem Cells International*.

[35] Al-Massri K. F., Ahmed L. A., El-Abhar H. S. (2019). Mesenchymal stem cells therapy enhances the efficacy of pregabalin and prevents its motor impairment in paclitaxel-induced neuropathy in rats: role of Notch 1 receptor and JAK/STAT signaling pathway. *Behavioural Brain Research*.

[36] Jensen T. S., Finnerup N. B. (2014). Allodynia and hyperalgesia in neuropathic pain: clinical manifestations and mechanisms. *The Lancet Neurology*.

[37] Alles S. R. A., Smith P. A. (2018). Etiology and pharmacology of neuropathic pain. *Pharmacological Reviews*.

[38] Chen G., Park C. K., Xie R. G., Ji R. R. (2015). Intrathecal bone marrow stromal cells inhibit neuropathic pain via TGF-*β* secretion. *The Journal of Clinical Investigation*.

[39] Chiang C. Y., Liu S. A., Sheu M. L. (2016). Feasibility of human amniotic fluid derived stem cells in alleviation of neuropathic pain in chronic constrictive injury nerve model. *PLoS One*.

[40] Costa G. M. F., de Oliveira A. P., Martinelli P. M., da Silva Camargos E. R., Arantes R. M. E., de Almeida-Leite C. M. (2016). Demyelination/remyelination and expression of interleukin-1*β*, substance P, nerve growth factor, and glial-derived neurotrophic factor during trigeminal neuropathic pain in rats. *Neuroscience Letters*.

[41] Merighi A. (2015). Targeting the glial-derived neurotrophic factor and related molecules for controlling normal and pathologic pain. *Expert Opinion on Therapeutic Targets*.

[42] Sarveazad A., Janzadeh A., Taheripak G., Dameni S., Yousefifard M., Nasirinezhad F. (2019). Co-administration of human adipose-derived stem cells and low-level laser to alleviate neuropathic pain after experimental spinal cord injury. *Stem Cell Research & Therapy*.

[43] Yu H., Fischer G., Ebert A. D., Wu H.-E., Bai X., Hogan Q. H. (2015). Analgesia for neuropathic pain by dorsal root ganglion transplantation of genetically engineered mesenchymal stem cells: initial results. *Molecular Pain*.

[44] Zor F., Deveci M., Kilic A. (2014). Effect of VEGF gene therapy and hyaluronic acid film sheath on peripheral nerve regeneration. *Microsurgery*.

[45] Lopes F. R. P., Lisboa B. C. G., Frattini F. (2011). Enhancement of sciatic nerve regeneration after vascular endothelial growth factor (VEGF) gene therapy. *Neuropathology and Applied Neurobiology*.

[46] Pereira Lopes F. R., Martin P. K. M., Frattini F. (2013). Double gene therapy with granulocyte colony-stimulating factor and vascular endothelial growth factor acts synergistically to improve nerve regeneration and functional outcome after sciatic nerve injury in mice. *Neuroscience*.

[47] Lee H. L., Oh J., Yun Y. (2015). Vascular endothelial growth factor-expressing neural stem cell for the treatment of neuropathic pain. *Neuroreport*.

[48] Mannelli L. D. C., Tenci B., Micheli L. (2018). Adipose-derived stem cells decrease pain in a rat model of oxaliplatin-induced neuropathy: role of VEGF-A modulation. *Neuropharmacology*.

[49] Hulse R. P., Beazley-Long N., Hua J. (2014). Regulation of alternative VEGF-A mRNA splicing is a therapeutic target for analgesia. *Neurobiology of Disease*.

[50] Selvaraj D., Gangadharan V., Michalski C. . W. (2015). A functional role for VEGFR1 expressed in peripheral sensory neurons in cancer pain. *Cancer Cell*.

[51] Kamp J., Van Velzen M., Olofsen E., Boon M., Dahan A., Niesters M. (2019). Pharmacokinetic and pharmacodynamic considerations for NMDA-receptor antagonist ketamine in the treatment of chronic neuropathic pain: an update of the most recent literature. *Expert Opinion on Drug Metabolism & Toxicology*.

[52] Zhou X. L., Zhang C. J., Peng Y. N., Wang Y., Xu H. J., Liu C. M. (2019). ROR2 modulates neuropathic pain via phosphorylation of NMDA receptor subunit GluN2B in rats. *British Journal of Anaesthesia*.

[53] Chen J., Li L., Chen S. R. (2018). The *α*2*δ*-1-NMDA receptor complex is critically involved in neuropathic pain development and gabapentin therapeutic actions. *Cell Reports*.

[54] Cohen S. P., Mao J. (2014). Neuropathic pain: mechanisms and their clinical implications. *BMJ*.

[55] Aiyer R., Mehta N., Gungor S., Gulati A. (2018). A systematic review of NMDA receptor antagonists for treatment of neuropathic pain in clinical practice. *The Clinical Journal of Pain*.

[56] Guo W., Chu Y. X., Imai S. (2016). Further observations on the behavioral and neural effects of bone marrow stromal cells in rodent pain models. *Molecular Pain*.

[57] Chen N. F., Huang S. Y., Chen W. F. (2013). TGF-*β*1 attenuates spinal neuroinflammation and the excitatory amino acid system in rats with neuropathic pain. *The Journal of Pain*.

[58] Tsuda M. (2018). Modulation of pain and itch by spinal glia. *Neuroscience Bulletin*.

[59] Mika J., Zychowska M., Popiolek-Barczyk K., Rojewska E., Przewlocka B. (2013). Importance of glial activation in neuropathic pain. *European Journal of Pharmacology*.

[60] Forouzanfar F., Amin B., Ghorbani A. (2018). New approach for the treatment of neuropathic pain: fibroblast growth factor 1 gene-transfected adipose-derived mesenchymal stem cells. *European Journal of Pain*.

[61] Huang X., Wang W., Liu X. (2018). Bone mesenchymal stem cells attenuate radicular pain by inhibiting microglial activation in a rat noncompressive disk herniation model. *Cell and Tissue Research*.

[62] Romero-Ramirez L., Wu S., de Munter J., Wolters E. C., Kramer B. W., Mey J. (2020). Treatment of rats with spinal cord injury using human bone marrow-derived stromal cells prepared by negative selection. *Journal of Biomedical Science*.

[63] Ji R.-R., Gereau R. W., Malcangio M., Strichartz G. R. (2009). MAP kinase and pain. *Brain Research Reviews*.

[64] Watanabe S., Uchida K., Nakajima H. (2015). Early transplantation of mesenchymal stem cells after spinal cord injury relieves pain hypersensitivity through suppression of pain-related signaling cascades and reduced inflammatory cell recruitment. *Stem Cells*.

[65] Li J., Deng G., Wang H. (2017). Interleukin-1*β* pre-treated bone marrow stromal cells alleviate neuropathic pain through CCL7-mediated inhibition of microglial activation in the spinal cord. *Scientific Reports*.

[66] Teng Y., Zhang Y., Yue S. (2019). Intrathecal injection of bone marrow stromal cells attenuates neuropathic pain via inhibition of P2X4R in spinal cord microglia. *Journal of Neuroinflammation*.

[67] Lin C.-H., Wu S.-H., Lee S.-S. (2017). Autologous adipose-derived stem cells reduce burn-induced neuropathic pain in a rat model. *International Journal of Molecular Sciences*.

[68] Shetty A. K., Bates A. (2016). Potential of GABA-ergic cell therapy for schizophrenia, neuropathic pain, and Alzheimer’s and Parkinson’s diseases. *Brain Research*.

[69] Gwak Y. S., Hulsebosch C. E. (2011). GABA and central neuropathic pain following spinal cord injury. *Neuropharmacology*.

[70] Isaacson J. S., Scanziani M. (2011). How inhibition shapes cortical activity. *Neuron*.

[71] Gwak Y. S., Tan H. Y., Nam T. S., Paik K. S., Hulsebosch C. E., Leem J. W. (2006). Activation of spinal GABA receptors attenuates chronic central neuropathic pain after spinal cord injury. *Journal of Neurotrauma*.

[72] Liu J., Wolfe D., Hao S. (2004). Peripherally delivered glutamic acid decarboxylase gene therapy for spinal cord injury pain. *Molecular Therapy*.

[73] Colloca L., Ludman T., Bouhassira D. (2017). Neuropathic pain. *Nature Reviews. Disease Primers*.

[74] François A., Low S. A., Sypek E. I. (2017). A brainstem-spinal cord inhibitory circuit for mechanical pain modulation by GABA and enkephalins. *Neuron*.

[75] Eaton M. J., Martinez M. A., Karmally S. (1999). A single intrathecal injection of GABA permanently reverses neuropathic pain after nerve injury. *Brain Research*.

[76] Mukhida K., Mendez I., McLeod M. (2007). Spinal GABAergic transplants attenuate mechanical allodynia in a rat model of neuropathic pain. *Stem Cells*.

[77] Fandel T. M., Trivedi A., Nicholas C. R. (2016). Transplanted human stem cell-derived interneuron precursors mitigate mouse bladder dysfunction and central neuropathic pain after spinal cord injury. *Cell Stem Cell*.

[78] Hwang I., Hahm S.-C., Choi K.-A. (2016). Intrathecal transplantation of embryonic stem cell-derived spinal GABAergic neural precursor cells attenuates neuropathic pain in a spinal cord injury rat model. *Cell Transplantation*.

[79] Tashiro S., Nishimura S., Shinozaki M. (2018). The amelioration of pain-related behavior in mice with chronic spinal cord injury treated with neural stem/progenitor cell transplantation combined with treadmill training. *Journal of Neurotrauma*.

[80] Lin W., Xu L., Zwingenberger S., Gibon E., Goodman S. B., Li G. (2017). Mesenchymal stem cells homing to improve bone healing. *Journal of Orthopaedic Translation*.

[81] Fu X., Liu G., Halim A., Ju Y., Luo Q., Song A. G. (2019). Mesenchymal stem cell migration and tissue repair. *Cells*.

[82] Janssens R., Struyf S., Proost P. (2018). The unique structural and functional features of CXCL12. *Cellular & Molecular Immunology*.

[83] Moser B., Loetscher P. (2001). Lymphocyte traffic control by chemokines. *Nature Immunology*.

[84] Baggiolini M. (1998). Chemokines and leukocyte traffic. *Nature*.

[85] Liekens S., Schols D., Hatse S. (2010). CXCL12-CXCR4 axis in angiogenesis, metastasis and stem cell mobilization. *Current Pharmaceutical Design*.

[86] Bai L., Wang X., Li Z. (2016). Upregulation of chemokine CXCL12 in the dorsal root ganglia and spinal cord contributes to the development and maintenance of neuropathic pain following spared nerve injury in rats. *Neuroscience Bulletin*.

[87] Liu Z. Y., Song Z. W., Guo S. W. (2019). CXCL12/CXCR4 signaling contributes to neuropathic pain via central sensitization mechanisms in a rat spinal nerve ligation model. *CNS Neuroscience & Therapeutics*.

[88] Hu Y., Chen W., Wu L., Jiang L., Qin H., Tang N. (2019). Hypoxic preconditioning improves the survival and neural effects of transplanted mesenchymal stem cells via CXCL12/CXCR4 signalling in a rat model of cerebral infarction. *Cell Biochemistry and Function*.

[89] Kowalski K., Kołodziejczyk A., Sikorska M. (2017). Stem cells migration during skeletal muscle regeneration - the role of Sdf-1/Cxcr 4 and Sdf-1/Cxcr 7 axis. *Cell Adhesion & Migration*.

[90] Berta T., Qadri Y., Tan P. H., Ji R. R. (2017). Targeting dorsal root ganglia and primary sensory neurons for the treatment of chronic pain. *Expert Opinion on Therapeutic Targets*.

[91] Chamberlain G., Fox J., Ashton B., Middleton J. (2007). Concise review: mesenchymal stem cells: their phenotype, differentiation capacity, immunological features, and potential for homing. *Stem Cells*.

[92] Prockop D. J., Kota D. J., Bazhanov N., Reger R. L. (2010). Evolving paradigms for repair of tissues by adult stem/progenitor cells (MSCs). *Journal of Cellular and Molecular Medicine*.

[93] Mendonça M. V., Larocca T. F., de Freitas Souza B. S. (2014). Safety and neurological assessments after autologous transplantation of bone marrow mesenchymal stem cells in subjects with chronic spinal cord injury. *Stem Cell Research & Therapy*.

[94] Vickers E. R., Karsten E., Flood J., Lilischkis R. (2014). A preliminary report on stem cell therapy for neuropathic pain in humans. *Journal of Pain Research*.

[95] Vaquero J., Zurita M., Rico M. A. (2018). Intrathecal administration of autologous mesenchymal stromal cells for spinal cord injury: safety and efficacy of the 100/3 guideline. *Cytotherapy*.

[96] Vaquero J., Zurita M., Rico M. A. (2018). Intrathecal administration of autologous bone marrow stromal cells improves neuropathic pain in patients with spinal cord injury. *Neuroscience Letters*.

[97] Levi A. D., Okonkwo D. O., Park P. (2018). Emerging safety of intramedullary transplantation of human neural stem cells in chronic cervical and thoracic spinal cord injury. *Neurosurgery*.

[98] Liu L., Hua Z., Shen J. (2017). Comparative efficacy of multiple variables of mesenchymal stem cell transplantation for the treatment of neuropathic pain in rats. *Military Medicine*.

[99] Van Pham P. (2016). Clinical trials for stem cell transplantation: when are they needed?. *Stem Cell Research & Therapy*.

[100] Kimmelman J., Federico C. (2017). Consider drug efficacy before first-in-human trials. *Nature*.

[101] Barker R. A., Carpenter M. K., Forbes S. (2018). The challenges of first-in-human stem cell clinical trials: what does this mean for ethics and institutional review boards?. *Stem Cell Reports*.

